# Hot Deformation Behavior and Microstructure Evolution of a Fe-Ni-Cr Based Superalloy

**DOI:** 10.3390/ma19010058

**Published:** 2025-12-23

**Authors:** Yan Wang, Tianyi Wang, Guohua Xu, Shengkai Gong, Ning Liu, Shusuo Li, Qiuyu Wang, Wenqi Guo, Biao Zhang

**Affiliations:** 1 Research Institute of Aero-Engine, Beihang University, Beijing 100191, China; wangyan20251120@163.com (Y.W.);; 2 AECC Chengdu Engine Co., Ltd., Chengdu 610503, China; 3 Taihang Laboratory, Chengdu 610213, China; 4 Gaona Aero Material Co., Ltd., Beijing 100081, China; 5 Fushun Special Steel Co., Ltd., Fushun 113001, China; 6 School of Materials Science and Engineering, University of Science and Technology Beijing, Beijing 100083, China

**Keywords:** GH2787 superalloy, hot deformation, microstructural evolution, DRX mechanism, processing maps

## Abstract

The present study systematically investigated the hot deformation behavior of GH2787 superalloy within the temperature range of 1060–1120 °C and strain rates of 0.1–10 s^−1^. An Arrhenius-type constitutive equation was developed that accurately predicts the flow behavior, and the calculated thermal deformation activation energy Q is 364,401.19 J/mol. The hot working map was constructed based on the dynamic material model, which identified two preferred processing regions with power dissipation efficiency exceeding 0.3, and no flow instability was observed across the entire parameter range. Microstructural analysis reveals that the extent of dynamic recrystallization significantly increases with rising temperature and strain rate. Discontinuous dynamic recrystallization (via grain boundary bulging nucleation) serves as the dominant recrystallization mechanism in GH2787 superalloy during hot deformation, while continuous dynamic recrystallization (via subgrain rotation and coalescence) acts as a synergistic auxiliary mechanism, jointly driving microstructural evolution. This study provides important theoretical foundations for optimizing the hot working processes of GH2787 superalloy.

## 1. Introduction

Ni-based superalloys serve as cornerstone materials for high-temperature critical components in the aerospace and energy industries, particularly for turbine disks, blades, and other essential parts. Their exceptional mechanical strength, outstanding creep resistance, and remarkable microstructural stability under extreme thermomechanical loads are vital for ensuring the efficiency, reliability, and safety of advanced engines [1,2,3]. Among these superalloys, GH2787 superalloy is widely utilized for high-temperature components such as turbine engine working blades operating at 700–800 °C and compressor blades serving at 500–700 °C, owing to its high strength coupled with good ductility, excellent fatigue resistance, and superior corrosion performance [4,5]. The in-service performance of these critical components is intrinsically linked to the microstructure developed during hot-forming processes like forging, rolling, or extrusion. These thermomechanical processing routes involve complex deformation under controlled temperatures and strain rates. The hot deformation behavior of superalloys is characterized by a series of competing metallurgical phenomena, including work hardening, dynamic recovery (DRV), and dynamic recrystallization (DRX) [6,7,8,9]. The interplay of these mechanisms dictates the evolution of grain size, phase distribution, and dislocation substructures, ultimately determining the final mechanical properties of the forged components. Consequently, a comprehensive understanding of their hot deformation characteristics is a prerequisite for optimizing process parameters, avoiding defects such as flow instability or cracking, and achieving a uniform, fine-grained microstructure.

In the published literature, research on the hot deformation behavior of Ni-based superalloys has established a systematic methodological framework. Numerous researchers have determined the optimal hot working window for superalloys by constructing precise constitutive equations and integrating dynamic material models (DMM) to construct processing maps, thereby providing theoretical guidance for practical production processes [2,10,11,12]. By integrating various techniques such as scanning electron microscopy (SEM), electron backscatter diffraction (EBSD), and transmission electron microscopy (TEM), the specific nucleation and growth mechanisms of DRX during hot deformation have been revealed. For instance, Le et al. [13] demonstrated that the DRX mechanism of SLM Inconel 718 alloy simultaneously involves discontinuous dynamic recrystallization (DDRX) and continuous dynamic recrystallization (CDRX) mechanisms during hot deformation. Li et al. [14] discovered that during the hot deformation of GH4698 superalloy, pre-existing Σ3 twins can promote DRX nucleation through DDRX and twin dynamic recrystallization (TDRX) mechanisms. Charpagne et al. [15] further identified a novel heterogeneous epitaxial recrystallization mechanism in René 65 superalloy, occurring at low strain levels before being superseded by DDRX processes. Liu et al. [16] revealed in the Haynes 214 alloy that DDRX was activated under all deformation conditions, while CDRX mechanism activity decreased with increasing temperature, most significantly at a strain rate of 1 s^−1^. Furthermore, Wang et al. [17] noted that the CDRX mechanism in Inconel 740 superalloy is more readily induced at lower deformation temperatures, higher strain rates, and moderate strain levels. Despite its significant industrial relevance, systematic studies on the hot deformation behavior of the GH2787 superalloy remain relatively limited. Critical aspects such as its constitutive relationships, DRX kinetics, identification of stable and unstable processing regions, and the evolution of specific precipitate phases during deformation require further elucidation.

Within this context, the present study aims to systematically investigate the hot deformation behavior of the GH2787 superalloy within practical ranges of temperature and strain rate. This work will focus on: (1) establishing a precise constitutive model that accurately describes the flow stress; (2) constructing processing maps to identify safe processing windows and instability regions; and (3) correlating deformation parameters with the resulting microstructural evolution, with special emphasis on DRX behavior. The findings are expected to provide a theoretical foundation and practical guidance for the design and optimization of thermomechanical processing parameters for GH2787 superalloy components.

## 2. Materials and Methods

The nominal chemical composition of the GH2787 superalloy used in the present study is shown in Table 1. The alloy was produced via vacuum induction melting (VIM) followed by electroslag remelting (ESR) to form an ingot. After homogenization treatment, the alloy was fabricated into 20 mm diameter bars. To obtain a similar initial microstructure prior to thermal deformation, the material was held at 1160 °C for 5 min. Cylindrical specimens with dimensions of 8 mm in diameter and 12 mm in height were then machined from the bar using wire electrical discharge machining (CHMER, Taichung City, Taiwan, China) for hot compression tests. All hot compression tests were conducted on a Gleeble-3500 thermomechanical simulator (Dynamic Systems Inc., El Segundo, CA, USA). Graphite foils were placed at both ends of the specimens to minimize the influence of friction on the stress state. A thermocouple was attached at the mid-height of the specimen to monitor the temperature in real time. During testing, the specimens were heated to temperatures ranging from 1060 °C to 1120 °C (at 20 °C intervals) at a rate of 10 °C/s and held for 5 min to ensure uniform temperature distribution. Subsequently, the specimens were axially compressed to a true strain of 0.7 at different strain rates (0.1, 1, 5, and 10 s^−1^) to systematically investigate the effects of temperature and strain rate on the hot deformation behavior and microstructural evolution of the alloy. Due to the high cost of thermal simulation experiments, a single test was conducted for all 16 process parameter combinations in this study. To ensure the reliability of critical data, repeatability tests were conducted at four representative parameter conditions (1060 °C, 1 s^−1^, 1080 °C, 10 s^−1^, 1100 °C, 1 s^−1^, 1100 °C, 10 s^−1^).

After compression, each specimen is sectioned along the compression axis to expose the longitudinal plane. The longitudinal plane was sequentially polished using SiC sandpaper ranging from 320 to 2000 grit, followed by mechanical polishing with a 2.5 μm diamond suspension to achieve a scratch-free mirror finish. After ultrasonic cleaning in ethanol and drying, the surface was further prepared by electrolytic polishing to remove any residual deformation layer and obtain a strain-free surface suitable for electron backscatter diffraction (EBSD) analysis. Electrolytic polishing was performed in a solution of 80 vol% CH_3_OH + 20 vol% H_2_SO_4_ at 10 V for 8 s. EBSD data were acquired from the central region of the cross-section parallel to the compression axis using a ZEISS ULTRA 55 field emission scanning electron microscope (Carl Zeiss AG, Oberkochen, Germany) equipped with an HKL Nordlys F+ EBSD detector (Oxford Instruments, Oxford, UK) at an acceleration voltage of 20 kV and a step size of 0.5 μm. For each sample under different conditions, three non-overlapping fields of view were selected for scanning, with each field covering an area of 280 × 210 μm^2^. The final reported statistical value represents the arithmetic mean of the measurements from these independent fields of view. The collected data were analyzed using Channel 5 and Aztec Crystal 2.1 software to obtain microstructural characteristic maps, including inverse pole figure (IPF), grain boundaries (GBs), kernel average misorientation (KAM), and grain orientation spread (GOS), under selected deformation conditions.

## 3. Results and Discussion

### 3.1. Microstructure Before Compression

**Figure 1:**
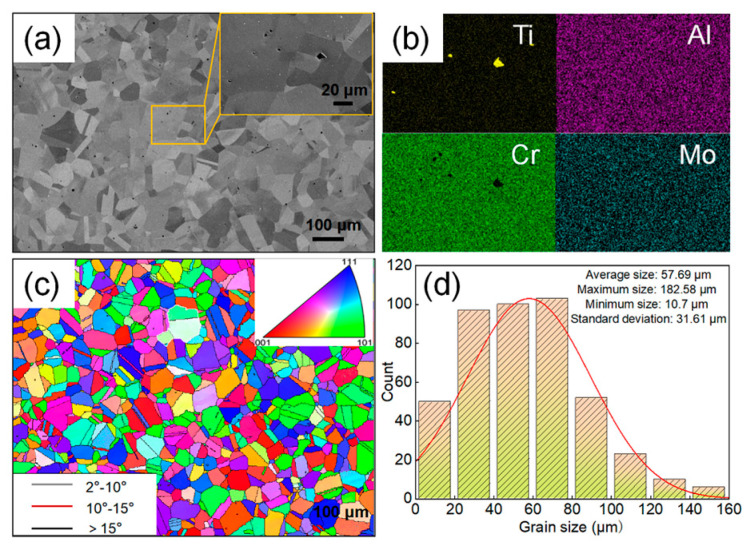
Microstructure of the GH2787 superalloy before compression: (**a**) SEM image, (**b**) SEM-EDS image, (**c**) IPF image, (**d**) Equivalent diameter distribution.

Figure 1 illustrates the initial microstructure of the GH2787 superalloy before compression. As shown in Figure 1a, the alloy exhibits a homogeneous equiaxed grain structure. Fine gray blocky and white granular secondary phases are dispersed within the grains (see inset in Figure 1a). EDS analysis (Figure 1b) indicates that the gray blocky particles are Ti-rich MC carbides, while the white granular phases are Cr-rich M_23_C_6_ carbides. The IPF map in Figure 1c reveals a random distribution of grain orientations, suggesting the absence of a pronounced crystallographic texture. Figure 1d presents the statistical analysis of the grain size distribution. The size distribution histogram exhibits a unimodal distribution. Further fitting of the grain size distribution using a normal curve demonstrates excellent agreement with experimental measurements, confirming that the size distribution is concentrated around the average grain size with no significant skewness. The calculated average value is approximately 57.69 μm, with a standard deviation of 31.61 μm, indicating a relatively narrow grain size distribution. This fine and uniform initial microstructure facilitates subsequent hot deformation by providing a high density of grain boundaries that promote mechanisms such as DRX.

### 3.2. Flow Stress

**Figure 2:**
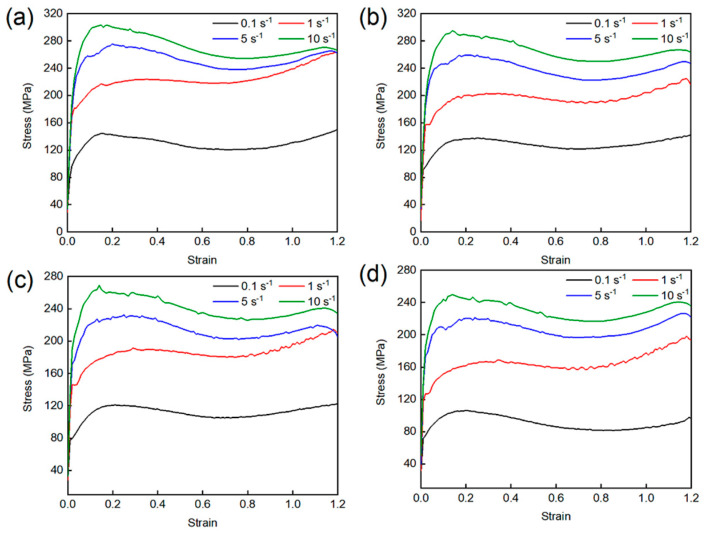
True stress–strain curves of GH2787 superalloy at: (**a**) 1060 °C, (**b**) 1080 °C, (**c**) 1100 °C and (**d**) 1120 °C.

Figure 2 presents the true stress–strain curves of GH2787 superalloy compressed at 1060, 1080, 1100, and 1120 °C. At the initial stage of deformation, dislocation density increases rapidly due to intensified dislocation motion. The resulting dislocation tangles and pile-ups impede further dislocation glide, leading to significant work hardening, which is reflected in the sharp rise in stress with strain. Once the dislocation density reaches a critical value, DRV and DRX are activated. These processes gradually mitigate the increase in dislocation density and weaken the work hardening effect, though work hardening remains dominant until the peak stress is attained. Beyond the peak stress, the softening effects induced by DRV and DRX become predominant. The dislocation density decreases progressively, reducing the flow stress required for continued deformation. When a dynamic balance is established between work hardening and softening, the flow stress fluctuates within a narrow range. At true strains exceeding 0.7, a slight upward trend is observed in the curves under certain conditions, which can be attributed to the deformation of newly formed DRX grains, leading to renewed work hardening.

**Figure 3:**
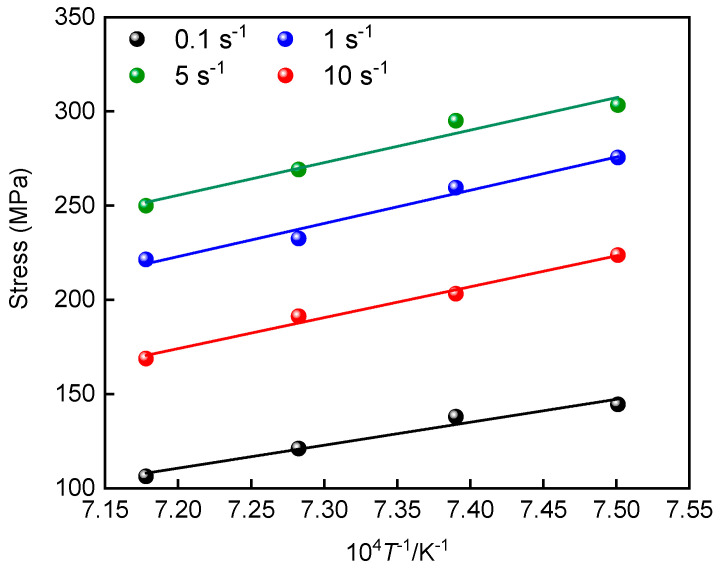
Variation in peak stress under various deformation conditions.

Moreover, at a given deformation temperature, the flow stress at a specific strain level increases with increasing strain rate, as shown in Figure 3. This is because higher strain rates reduce the time available for DRV and DRX to fully proceed, thereby limiting their softening effects. Meanwhile, the rapid generation of dislocations within a short period enhances work hardening, resulting in higher flow stresses. Conversely, increasing the deformation temperature leads to a decrease in flow stress. On one hand, higher temperatures facilitate dislocation unpinning and activation of additional slip systems, thereby alleviating work hardening. On the other hand, increased atomic diffusion and grain boundary mobility at elevated temperatures enhance DRX nucleation and growth, strengthening the overall softening response.

### 3.3. Arrhenius-Type Constitutive Equation

Typically, the relationship between flow stress and deformation temperature and strain rate during hot deformation can be effectively modeled using an Arrhenius-type constitutive equation, which can be expressed as [18]:

(1) $$\dot{\varepsilon }=A{\left[\sinh\left(\alpha \sigma \right)\right]}^{n}\exp\left(\frac{–Q}{RT}\right)$$

where *σ* is the stress (MPa), $\dot{\varepsilon }$ is the strain rate (s^−1^), *Q* is the hot deformation activation energy of the alloy (J·mol^−1^), *T* is the deformation temperature (K) and *R* is the universal gas constant (8.314 J·mol^−1^·K^−1^). *A* and *α* are the material coefficients. Based on the stress level, Equation (1) can be classified into the following specific forms [19]:

(2) $$\dot{\varepsilon }=A_{1}\sigma^{n_{1}}\exp\left(\frac{–Q}{RT}\right)\;\;\alpha \sigma \;<\;0.8$$

(3) $$\dot{\varepsilon }=A_{2}\exp\left(\beta \sigma \right)\exp\left(\frac{–Q}{RT}\right)\;\;\alpha \sigma >1.2$$

To linearize the relationship for subsequent analysis, the natural logarithm is taken on both sides of Equations (1)–(3), obtaining Equations (4)–(6):

(4) $$\ln\dot{\varepsilon }=\ln A+n\ln\left[\sinh\left(\alpha \sigma \right)\right]-\frac{Q}{RT}$$

(5) $$\ln\dot{\varepsilon }=\ln A_{1}+n_{1}\ln\sigma -\frac{Q}{RT}$$

(6) $$\ln\dot{\varepsilon }=\ln A_{2}+\beta \sigma -\frac{Q}{RT}$$

Under the assumption that *Q* is independent *T*, the peak stresses from Figure 2 and the corresponding $\dot{\varepsilon }$ were substituted into Equations (4)–(6), the relationship between material constants can be expressed as Equation (7):

(7) $$\beta =\frac{\partial \ln\dot{\varepsilon }}{\partial \sigma };\;n_{1}=\frac{\partial \ln\dot{\varepsilon }}{\partial \ln\sigma };\;n=\frac{\partial \ln\dot{\varepsilon }}{\partial \ln\left[\sinh(\alpha \sigma )\right]}$$

**Figure 4:**
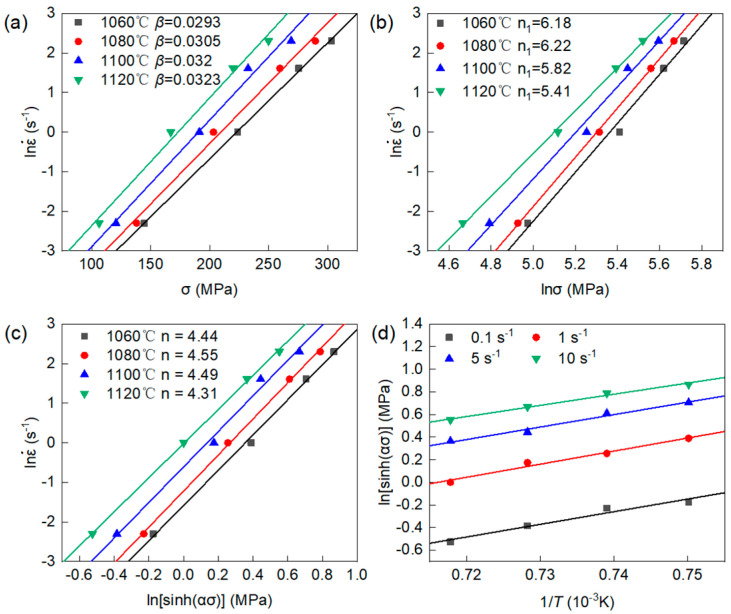
Fitting parameters for the Arrhenius-type constitutive equation: (**a**) $ln\dot{\varepsilon }$-σp, (**b**) $ln\dot{\varepsilon }$-lnσp, (**c**) $ln\dot{\varepsilon }$-$ln\left[sinh\left(\alpha \sigma \right)\right]$, (**d**) $ln\left[sinh\left(\alpha \sigma \right)\right]$-1/T.

The values of the material constants *n*_1_, *β*, and *n* were determined from the average slopes of the linear relationships presented in Equation (7), and the results are shown in Figure 4a,b. The final values were determined as *n*_1_ = 6.4199, *β* = 0.0335, *n* = 4.8378, *α* = *β*/*n*_1_ = 0.0052 and *A* = 5.9544 × 10^13^. The average activation energy *Q* can be expressed as $Q=R{\left|\frac{\partial \ln\dot{\varepsilon }}{\partial \ln\left[\sinh\left(\alpha \sigma \right)\right]}\right|}_{T}{\left|\frac{\partial \ln\left[\sinh\left(\alpha \sigma \right)\right]}{\partial \left(1/T\right)}\right|}_{\dot{\varepsilon }}$ through the differential transformation of Equation (4) and *Q* = 364,401.19 J/mol. This value is significantly lower than that of comparable Fe-Ni-Cr-based high-temperature alloys, such as GH4169 (*Q* ≈ 450,977 J/mol) [20] and GH2132 (*Q* ≈ 458,388 J/mol) [21]. This difference is attributed to the coupling effects of alloy composition. By applying the exponential relationship between activation energy and elemental content established by Bi et al. [22], the *Q*-value for GH2787 superalloy is estimated to be 344,100 J/mol. This value is consistent with the results obtained from constitutive equation calculations.

Furthermore, the average value can be determined by linear fitting the peak stress to the corresponding strain rate, as shown in Figure 4c,d. Ultimately, based on the values of *A*, *α*, *n*, and *Q* obtained from the fit to the experimental data, Equation (3) can be expressed as follows:

(8) $$\dot{\varepsilon }=5.9544\times 10^{13}{\left[\sinh\left(0.0052\sigma \right)\right]}^{4.8378}\exp\left(\frac{–364,401.19}{RT}\right)$$

The accuracy of Equation (8) can be verified by using the temperature-compensated strain rate parameter Z (Zenner-Holoman), which can be expressed as [23]:

(9) $$Z=\dot{\varepsilon }\exp\left(\frac{Q}{RT}\right)=A{\left[\sinh\left(\alpha \sigma \right)\right]}^{n}$$

**Figure 5:**
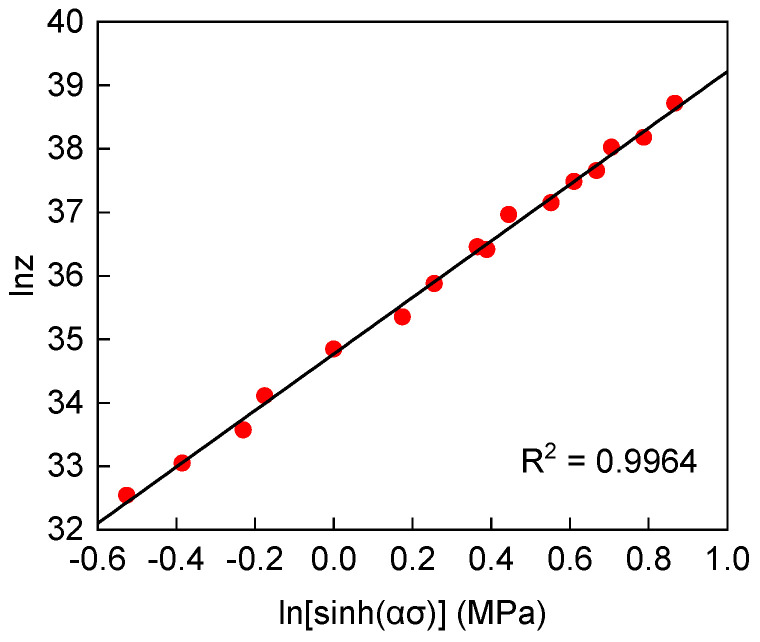
Relationship between peak stress and Z during hot deformation of GH2787 superalloy.

By performing logarithmic transformation on Equation (9) and conducting linear regression analysis, the relationship between the peak stress and the Z parameter was established, as shown in Figure 5. Under the condition of 70% engineering strain, the linear regression yielded a correlation coefficient (R^2^) as high as 99.64%, which strongly validates the high predictive accuracy and reliability of the developed constitutive equation.

### 3.4. Processing Maps for GH2787 Superalloy

**Figure 6:**
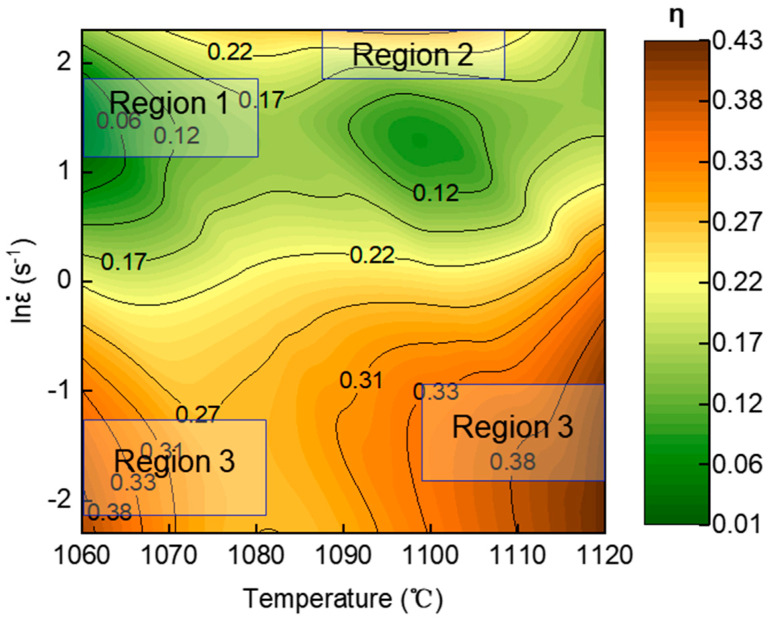
Processing maps for GH2787 superalloy at 1.2 strain.

To optimize the hot workability of the GH2787 superalloy, a processing map was constructed in this section following the DMM by Prasad et al. [24,25,26]. This map enables the systematic identification of energy dissipation characteristics and flow instability regions under various deformation parameters (temperature and strain rate), thereby clearly delineating the safe and unstable processing domains. The results provide direct guidance for selecting the optimal hot deformation parameters in industrial production. Figure 6 presents the hot processing map of GH2787 superalloy at 1.2 true strain, where the contour lines and color shading collectively reflect the distribution of the power dissipation efficiency factor (*η*). It can be observed that across the entire range of hot deformation parameters, the *η* values fall within 0.2–0.33. This variation reflects differences in microstructural evolution mechanisms under varying deformation conditions, and is closely related to the evolution of DRX behavior, which will be discussed in detail in the next section. It is generally accepted that regions with higher *η* values (typically *η* > 0.3) correspond to better hot workability [27]. For the GH2787 superalloy, two regions with high *η* values are identified, with the *η* value remaining consistently above 0.3. These regions correspond to the following processing windows: 1060–1070 °C, 0.01–0.6 s^−1^, and 1088–1120 °C, 0.01–1 s^−1^. Notably, the *η* value only reflects the trend of energy dissipation, and the optimal processing window still requires further verification through microstructural observation. Moreover, no instability regions are observed within the entire experimental parameter range, indicating that GH2787 superalloy exhibits excellent hot workability under these conditions, with a low risk of instability-induced cracking. This provides a safe processing window for the industrial hot working of this alloy.

### 3.5. Microstructural Evolution

**Figure 7:**
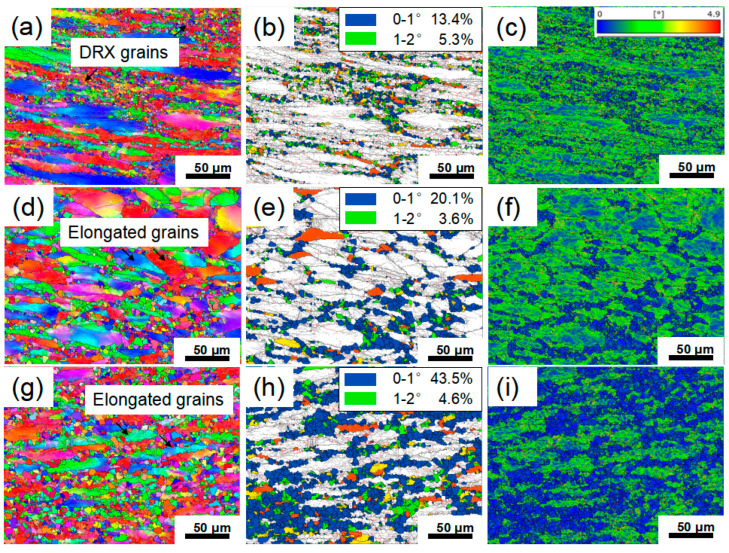
Representative microstructure of Region 1: (**a**–**c**) 1060 °C, 1 s^−1^ deformation condition; (**d**–**f**) 1060 °C, 5 s^−1^ deformation condition, (**g**–**i**) 1080 °C, 1 s^−1^ deformation condition, (**a**,**d**,**g**) IPF map, (**b**,**e**,**h**) GOS map, (**c**,**f**,**i**) KAM map.

**Figure 8:**
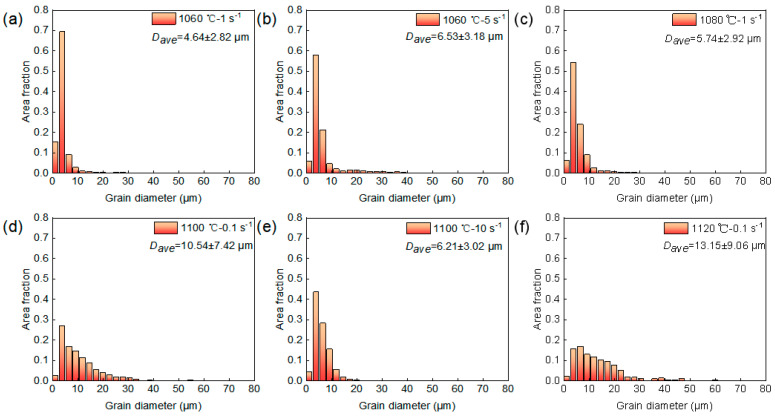
Statistical results of average grain size under different deformation conditions: (**a**) 1060 °C, 1 s^−1^; (**b**) 1060 °C, 5 s^−1^; (**c**) 1080 °C, 1 s^−1^; (**d**) 1100 °C, 0.1 s^−1^; (**e**) 1100 °C, 10 s^−1^; (**f**) 1120 °C, 0.1 s^−1^.

**Figure 9:**
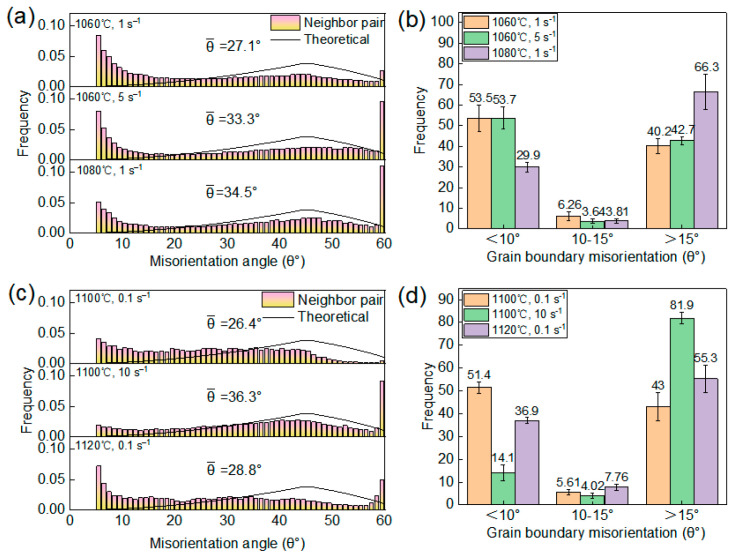
(**a**,**c**) Distribution of grain boundary misorientation at different deformation conditions; (**b**,**d**) corresponding to the fractions of LAGBs, MAGBs and HAGBs in (**a**,**c**), respectively.

**Figure 10:**
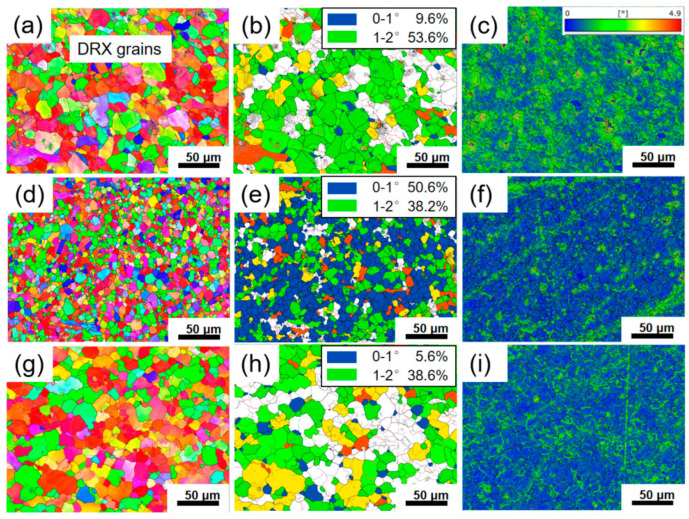
Representative microstructure of Region 2 and 3: (**a**–**c**) 1100 °C, 0.1 s^−1^ deformation condition; (**d**–**f**) 1100 °C, 10 s^−1^ deformation condition, (**g**–**i**) 1120 °C, 0.1 s^−1^ deformation condition, (**a**,**d**,**g**) IPF map, (**b**,**e**,**h**) GOS map, (**c**,**f**,**i**) KAM map.

To systematically investigate the correlation between processing parameters and microstructure, three representative regions were selected for EBSD analysis based on variations in *η* values within the processing map, marked as Regions 1 to 3. Specifically, Region 1 represents the low-dissipation region (*η* < 0.2), Region 2 represents the medium-dissipation region (0.3 > *η* > 0.2), and Region 3 corresponds to the high-dissipation region (*η* > 0.3). The corresponding results are shown in Figure 7, Figure 8, Figure 9 and Figure 10. The grain boundary delineation criteria in all IPF maps were consistent with those in Figure 1 to ensure comparability. Furthermore, to visually represent the degree of recrystallization in the selected deformation conditions, GOS maps were employed to characterize the local strain distribution, thereby enabling the identification of recrystallized and deformed structures. In this study, a threshold of 2° was used to identify DRX grains, and the volume fraction of DRX grains was quantitatively evaluated from the GOS maps. In addition, KAM maps were used to characterize the local plastic strain and dislocation density during hot deformation, where blue regions indicate low KAM values and green regions represent high KAM values.

Figure 7 shows the EBSD results for Region 1. It can be observed that the microstructure in this region is mainly composed of elongated deformed grains and fine necklace-like grains formed along the boundaries (Figure 7a). These fine grains exhibit GOS values below 2° and correspond to low KAM values (Figure 7b,c), which confirms that those are DRX grains. Comparing Figure 7a–f further reveal subtle variations induced by changes in strain rate. As the strain rate increases from 1 s^−1^ to 5 s^−1^, the volume fraction of DRX grains increases from 18.7 ± 3.6% to 23.7 ± 4.8%. This is attributed to the rapid accumulation of dislocations within a short time at higher strain rates, leading to higher local orientation gradients and stored energy inside the deformed grains. This reduces the critical nucleation size for DRX and enhances the driving force for nucleation, thereby promoting the formation of DRX grains and resulting in a higher volume fraction of DRX [28]. Similarly, comparing Figure 7a–c,g–i illustrate the effect of deformation temperature. When the temperature increases from 1060 °C to 1080 °C, the DRX volume fraction increases significantly from 18.7 ± 3.6% to 48.1 ± 5.2%. This trend is attributed to two main factors: on one hand, elevated temperature enhances atomic diffusion, accelerating grain boundary migration and the growth of recrystallized grains; on the other hand, enhanced thermal activation facilitates dislocation climb and cross-slip, making the nucleation and growth of DRX more favorable [29]. Figure 8 shows the distribution of grain sizes after deformation. As illustrated in Figure 8a–c, the average grain size in Region 1 predominantly ranges between 4.64–6.53 μm. Compared to the initial size (57.69 μm), DRX nucleation and refinement significantly reduced the overall average grain size. Moreover, DRX is accompanied by the annihilation of low-angle grain boundaries (LAGBs) and the formation of high-angle grain boundaries (HAGBs). Figure 9a,b show the distribution of misorientation angle within Region 1. As the temperature increases, the average misorientation angle increases from 27.1° to 34.5°; whereas as the strain rate increases, the average misorientation angle rises from 27.1° to 33.3°. Meanwhile, the proportion of LAGBs decreased from 53.5% and 53.7% to 29.9%, respectively. Under high temperature or high strain rate conditions, the DRX process becomes more active, generating HAGBs by consuming subgrain structures (LAGB networks). This drives an increase in the average dislocation orientation angle and shifts the distribution toward higher angles overall.

Figure 10 shows the EBSD results for Regions 2 and 3. As can be observed that although the *η* value at 1100 °C, 10 s^−1^ is below 0.3, the original elongated grain microstructure has been completely replaced by uniform equiaxed DRX grains, confirming that the DRX process has been completed. Notably, during deformation at 1120 °C, 0.1 s^−1^ and 1100 °C, 0.1 s^−1^, the GOS values of DRX grains were predominantly distributed between 1–2°, with KAM images revealing locally high KAM stripes along certain DRX grain boundaries (Figure 10c,f,i). This indicates that newly formed DRX grains underwent subsequent plastic deformation, leading to renewed dislocation pile-ups at these boundaries. This phenomenon is further verified by Figure 9c,d. Specifically, compared to the deformation conditions of 1100 °C, 10 s^−1^, the average misorientation angle decreased from 36.3° to 26.4° and 28.8° under the conditions of 1100 °C and 0.1 s^−1^ and 1120 °C and 0.1 s^−1^, respectively. The corresponding proportion of HAGBs also decreased from 81.9% to 43.0% and 55.3%. This microscopic mechanism is consistent with the secondary hardening phenomenon observed in the true stress–strain curves above 0.7 true strain in Figure 2. Furthermore, comparing the DRX grain sizes under different deformation conditions reveals that the grain size obtained at 1100 °C, 10 s^−1^ is significantly finer than those at 1100 °C, 0.1 s^−1^ and 1120 °C, 0.1 s^−1^ (Figure 8d–f). This can be explained by the dual effect of strain rate on the DRX process; the higher strain rate (10 s^−1^) markedly enhances dislocation accumulation, thereby increasing the DRX nucleation rate. However, it concurrently shortens the available time for grain growth. The interplay of these two factors ultimately results in a refined DRX grain structure.

### 3.6. DRX Mechanism

Based on the flow behavior and EBSD statistical results, it is clear that GH2787 superalloy undergoes varying degrees of DRX during hot deformation, with this process strongly dependent on changes in temperature and strain rate. Specifically, both DRX significantly enhanced with increasing deformation temperature and strain rate. However, the specific DRX mechanism during the hot deformation process of this alloy requires further clarification.

**Figure 11:**
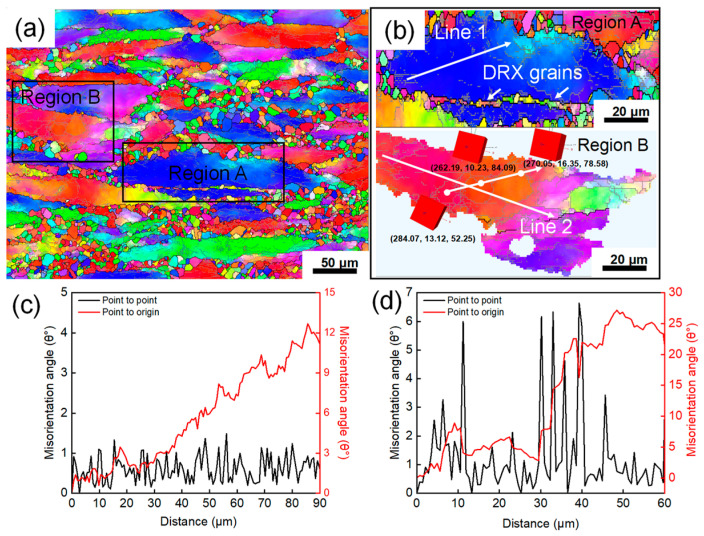
Microstructure under 1080 °C, 5 s^−1^ deformation condition: (**a**) IPF map and (**b**) grain boundary map, (**c**,**d**) Misorientation profiles of L1 and L5 in (**b**), respectively.

Figure 11a,b show the EBSD analysis for Region 1 deformed at 1080 °C and 5 s^−1^, revealing a partially recrystallized microstructure, which is consistent with the prediction of the processing map. As previously mentioned, in Ni-based superalloys, the DRX mechanism primarily involves two categories: one is DDRX, characterized by grain boundary bulging and subsequent migration, encompassing DRX nucleation and grain growth. The other is CDRX, characterized by subgrain rotation, accompanied by the gradual transformation of low-angle grain boundaries (LAGBs) into high-angle grain boundaries (HAGBs). As shown in Region A of Figure 10b, the original deformed grain boundaries exhibit a serrated morphology, indicating the occurrence of strain-induced grain boundary migration. Fine equiaxed grains are distributed around these serrated boundaries, features typically recognized as signals for the onset of DDRX [30]. Furthermore, within Region B, apparent color gradation can be observed inside some deformed grains, indicating that the crystal orientation within these grains is undergoing a gradual transition, a phenomenon usually associated with CDRX [13].

Constructing local misorientation profiles parallel and perpendicular to grain boundaries is an effective method for identifying these two DRX mechanisms. When the cumulative misorientation within a deformed grain exceeds 15°, and the point-to-point misorientation shows peaks surpassing 2°, it is defined as the CDRX mechanism characterized by progressive subgrain rotation [31]. Conversely, if these conditions are not met, it is primarily defined as the DDRX mechanism characterized by grain boundary bulging nucleation. Figure 11c,d show the misorientation profiles along lines L1 and L2 in Regions A and B, respectively. It can be seen that the cumulative misorientation (point-to-origin) for L1 increases rapidly with distance, exceeding 15° at 35 μm and reaching a peak of 27.17° at 50 μm. Additionally, within the range of 10–50 μm, several peaks above 2° are observed in the point-to-point misorientation profile of L1, indicating the well-developed substructure surrounded by LAGBs. This evidence confirms the activation of CDRX characterized by progressive subgrain rotation. In contrast, the cumulative misorientation for L2 remains around 12° over 90 μm, and its point-to-point misorientation is far below 2°. Therefore, DDRX nucleation predominates in the area corresponding to L2.

Furthermore, the DRX process is accompanied by a transition characterized by a decrease in LAGBs and an increase in HAGBs. Mechanistically, in DDRX, which proceeds primarily through the migration of pre-existing HAGBs, the transition from LAGBs to HAGBs is essentially instantaneous. In contrast, CDRX involves the gradual transformation of LAGBs into HAGBs, a process that increases the proportion of medium-angle grain boundaries (MAGBs, typically defined with misorientation angles around 10–15°) [32,33]. Consequently, changes in the fraction of MAGBs are generally considered an indicator of the activation of the CDRX mechanism. The statistical results of grain boundary misorientation distributions in Figure 8b,d reveal that the fraction of MAGBs decreases with increasing deformation temperature, while variations in strain rate do not cause significant changes in the MAGBs fraction. This confirms that the CDRX mechanism is more active at lower temperatures, and its activity is less sensitive to changes in strain rate. Although the presence of MAGB and variations with temperature/strain rate confirm the activation of the CDRX mechanism, the consistently low proportion (approximately 2%) indicates limited efficiency in forming new grains via subgrain progressive rotation. Therefore, combined with the pronounced protrusion of primary grain boundaries (Figure 6), it can be concluded that DDRX is the dominant recrystallization mechanism, while the CDRX mechanism is considered a supplementary mechanism.

## 4. Conclusions

The present study systematically investigated the flow behavior, microstructural evolution, and dynamic recrystallization mechanisms of GH2787 superalloy under thermal compression conditions ranging from 1060 to 1120 °C and strain rates of 0.1 to 10 s^−1^. The main conclusions obtained are as follows:

(1) An Arrhenius-type constitutive model was established for GH2787 superalloy under high-temperature deformation conditions, demonstrating accurate prediction of flow stress behavior across different temperatures and strain rates, and can be expressed as:
  $$\dot{\varepsilon }=5.9544\times 10^{13}{\left[\sinh\left(0.0052\sigma \right)\right]}^{4.8378}\exp\left(\frac{–364,401.19}{RT}\right)$$
(2) The processing map constructed based on the DMM model reveals two domains with power dissipation efficiency greater than 0.3, corresponding to the following deformation parameter ranges: 1060–1068 °C, 0.01–0.6 s^−1^, and 1088–1120 °C, 0.01–0.6 s^−1^. Notably, no flow instability regions were observed throughout the experimental parameter range.
(3) The extent of DRX is significantly enhanced with increasing deformation temperature and strain rate. During the hot deformation, both DDRX and CDRX mechanisms are simultaneously active. DDRX dominates the recrystallization process through grain boundary bulging and nucleation, while CDRX participates in microstructural evolution through subgrain rotation and coalescence as auxiliary mechanisms.

## Figures and Tables

**Table 1:** Composition of GH2787 superalloy (wt.%).

C	Ni	Cr	W	Mn	Si	Al	Ti	Fe
0.06	35.6	15.5	3.23	0.14	0.14	1.23	2.94	Bal.

## Data Availability

The original contributions presented in this study are included in the article. Further inquiries can be directed to the corresponding authors.
